# Application of the quality of recovery-40 questionnaire to evaluate the effectiveness of enhanced recovery after surgery protocols in gastric cancer

**DOI:** 10.1007/s13304-023-01719-w

**Published:** 2024-01-21

**Authors:** Yeyang Chen, Siyu Liu, Bopei Li, Rujing Lin, Weikun Lai, Dejun Liu, Zhen Wang, Jinlu Liu, Xingan Qin, Xianghua Wu, Jiehua Li, Kui Jia, Junqiang Chen

**Affiliations:** 1 Department of Gastrointestinal Surgery, The First Affiliated Hospital of Guangxi Medical University, 6 Shuangyong Road, Nanning, 530021 China; 2 Guangxi Key Laboratory of Enhanced Recovery After Surgery for Gastrointestinal Cancer, Guangxi, China; 3 Department of Thyroid and Breast surgery, The First People’s Hospital of Yulin, Yulin, China

**Keywords:** Enhanced recovery after surgery, The Quality of Recovery-40 Questionnaire, Gastric cancer, Patient-reported, Effectiveness

## Abstract

Patient reported outcomes is currently considered to be an important supplement to evaluate the effectiveness of enhanced recovery after surgery (ERAS) clinical practice. The Quality of Recovery-40 Questionnaire (QoR-40) is one of the most frequently used and validation tool to assess the subjective feelings of quality of life after surgery. The present study aimed to use the QoR-40 to evaluate the effectiveness of ERAS protocols in gastric cancer from the perspective of patient-reported quality of recovery. The study was designed as a prospective, non-randomized clinical trial, conducted in a single center. Patients in our hospital who were scheduled to undergo radical surgery for gastric cancer were divided into ERAS group and control group (Contr group). The QoR-40 were administered one day before surgery (Baseline) and on postoperative day 1, 3, 6, and 30. The difference in QoR-40 scores between the ERAS and Contr groups was compared by repeated-measures ANOVA. A total of 200 patients completed the study, including 100 patients in the ERAS group and 100 patients in the Contr group. The Baseline time point QoR-40 scores of the ERAS and Contr groups were 179.68 ± 14.46 and 180.12 ± 17.12, respectively, and no significant difference was noted between the two groups (p = 0.845). The postoperative QoR-40 score of the ERAS group was significantly higher than that of the Contr group, and the difference was statistically significant (p = 0.006). This study demonstrated that, in terms of patient-reported quality of recovery, the postoperative recovery effect of ERAS protocols in gastric cancer is significantly better than that of the traditional treatment model.

## Introduction

Globally, gastric cancer (GC) is the fifth most common malignant tumor in terms of new incidence each year and is the fourth leading cause of cancer-related deaths [1]. The incidence of GC is considerably regional, and East Asia, including China, is reported to have the highest incidence of GC [2]. According to the statistical data, the 5-year overall survival rate of patients with GC in China in 2015 was 35.1% and that of patients with GC in the United States in 2014 was 33.1% [3, 4]. Treatment methods for GC include surgery, chemotherapy, radiotherapy, and targeted therapy. At present, surgery is the primary method of treatment for GC [5, 6].

Enhanced recovery after surgery (ERAS) is a comprehensive management plan for the perioperative period combined with evidence-based medicine. The primary goal of ERAS is to reduce trauma and stress. ERAS adopts a series of optimized measures in the perioperative period to promote rapid postoperative recovery, shorten the average length of hospital stay, and reduce hospitalization cost without affecting the incidence of postoperative complications. Therefore, while evaluating the effects of ERAS, researchers often use objective indicators from doctor-reported outcomes, such as average hospital stay, hospitalization cost, and postoperative complications, to evaluate its effectiveness [7, 8]. Studies on the effectiveness of ERAS, however, should include not only the objective data but also the subjective feelings of patients. In recent years, “patient-reported outcomes (PROs),” which are based on the concept of the bio-psycho-social medicine model, have been used to evaluate the effects of ERAS. The Quality of Life Assessment questionnaire is the most commonly used PRO tool.

The Quality of Recovery-40 Questionnaire (QoR-40)is a commonly used patient-rated quality of life questionnaire. It was developed and validated by Dr. Myles in Australia in 2000 [9]. It is mainly used to assess the early postoperative recovery of quality of life after general anesthesia and surgery. At present, QoR-40 has been validated and used in various countries and has been successfully used to evaluate the quality of recovery after different surgical methods or anesthesia [10–14]. Our previous study reported the development of the official Chinese version of QoR-40 (QoR-40C) and confirmed that it has good reliability, validity, and responsiveness and can be used to evaluate the quality of recovery of surgical patients [15].

In the present study, patients undergoing radical GC surgery were selected as the research population. The QoR-40 was used to evaluate the effectiveness of ERAS protocols in GC by comparing the ERAS and traditional intervention model and to determine the appropriate time to discharge from the perspective of patient-reported quality of recovery.

## Methods

### Study population

This study was approved by the Ethics Committee of the First Affiliated Hospital of Guangxi Medical University [Approval Number: 2020 (KY-E-078)], and all patients participating in this study were required to sign written informed consent. The study was designed as a prospective, non-randomized clinical trial, conducted in a single center in the First Affiliated Hospital of Guangxi Medical University. This study included patients who underwent radical surgery for GC from August 2019 to February 2021. During this period, patients were continuously recruited into the trial and divided into ERAS group and Contr group according to their wishes. The inclusion criteria were as follows: (1) the ability to take care of oneself, engage in light physical activity, and eat using the mouth; (2) organ function is sound or is compensated; (3) possibility to undergo D2 radical resection; (4) American Society of Anesthesiologists (ASA) grade ≤ III; (5) the patient and family members agree to participate in this project after being informed. The exclusion criteria were as follows: (1) no possibility to undergo D2 radical surgery; (2) ASA grade > III or advanced age (≥ 80 years old); (3) poor comprehension ability; (4) psychiatric/central nervous system disorders, history of alcohol or drug addiction, or presence of severe underlying diseases that prevent the subjective completion of the QoR-40. The included patients were divided into the ERAS group and the control group (Contr group). Patients were required to complete the QoR-40 on one day before surgery (Baseline) and on postoperative day (POD) 1, 3, 6, and 30. The ERAS group received ERAS protocol intervention, and the Contr group received traditional protocol intervention. The sample size was calculated as follows: (1) the pre-collection data of 30 patients were analyzed by PASS software version 15.0.5 and (2) the significance level was set at α = 0.05 (two-sided), and the power of test was set as 1 − β = 0.90. Based on the calculation, each group required a sample size of N = 83, assuming that the withdrawal rate was 10%, each group required at least 93 participants. Hence, considering that the study had two groups, a total of at least 186 patients needed to be included in this study.

#### Perioperative management protocols

The ERAS protocols of our center have been developed by referring to the “Consensus guidelines for enhanced recovery after gastrectomy Enhanced Recovery After Surgery (ERAS^®^) Society recommendations” [16] and are based on the five core elements proposed by the proponent of the ERAS concept, Professor Kehle [17]. Together with our center’s experience on clinical practice of ERAS, we formulated ERAS perioperative management protocols containing 22 items (Table 1).

**Table 1:** Perioperative management protocols for the ERAS and control groups

Perioperative management	Control group	ERAS group
*Admission*	Preoperative education No smoking and no alcohol consumption, lung function training if necessary	Preoperative education No smoking and no alcohol consumption, lung function training if necessary
*Preoperative*		
Preoperative nutritional support	For those with obvious malnutrition, take nutritional powder or enteral nutrient solution orally before surgery, and supplement with parenteral nutrition if necessary	For those with obvious malnutrition, take nutritional powder or enteral nutrient solution orally before surgery, and supplement with parenteral nutrition if necessary
Fasting before surgery	Fasting for 12 h before surgery and no water consumption for 6 h	Oral energy solution 10 h and 2 h before surgery
Bowel preparation	Routine oral laxative one day before surgery	Nonroutine mechanical bowel preparation
Preoperative drug application	Unconventional use of diazepam for sedation	Unconventional use of diazepam for sedation
Antibacterial prevention and skin preparation	Prepare the skin the night before surgery, and use antibiotics 30 min before the surgery	Prepare the skin the night before surgery, and use antibiotics 30 min before the surgery
*Intraoperative*		
Anesthesia protocol	Traditional anesthesia protocol	Optimize anesthesia protocol (general anesthesia combined with epidural anesthesia)
Type of surgery	No special requirements	Priority is given to laparoscopic or robotic surgery or short incision surgery
Body temperature intervention	No special intervention required unless the patient is hypothermic	Use heaters and other equipment to maintain stable body temperature
Preventive analgesia	No preventive analgesia	Apply TAP and PCA, local infiltration anesthesia with ropivacaine before abdominal closure
Drainage tube	Routine use of three abdominal drainage tubes (under the liver, pelvis, and splenic fossa)	Avoid using abdominal drainage tube; if used, remove it early after surgery
Liquid management	Do not deliberately limit the amount of liquid	Strict fluid control, preferentially select vasoconstrictor drugs to control blood pressure
Gastrointestinal decompression	Routine gastrointestinal decompression before surgery	Nonroutine gastrointestinal decompression before surgery
*Postoperative*		
Postoperative antiemetic	Use antiemetics only when the patient needs	Routine preventive use of antiemetic drugs
Postoperative analgesia	Indwelling PCA, providing analgesics only when the patient needs, and performing VAS scores daily	Indwelling PCA,multimodal analgesia from the day after surgery, and performing VAS scores daily
Catheter and gastrointestinal decompression	Routine indwelling for 3 days or more; to decide whether to remove according to the drainage volume and the nature of the drainage fluid	Remove the nasogastric tube and drainage tube as soon as possible after the operation, and remove the urinary tube on the first day after the operation
Early postoperative eating	After the operation, take oral food according to the removal of the gastric tube and the patient's wish	From the first day after the operation, the patient is instructed to take saline and gradually transition to clear liquid, liquid, semi-liquid, and pharmacological nutrients if necessary
Blood sugar control	Measure blood glucose only when abnormal blood glucose level is suspected	Measure blood glucose level three times a day, and perform timely intervention for patients with abnormal blood glucose level
Promote recovery of bowel function	No special treatment; wait for the patient to recover on their own	Lactulose oral liquid 15 ml/time, 2–3 times a day
Postoperative activities	Decide the time of getting out of bed according to the patient's wish	On the first day after surgery, the patient should sit up for at least 6 h. From the second day onwards, the patient should get out of bed for 2 h, and the amount of exercise should gradually increase
Quality of life assessment	Use QoR-40 to assess the quality of life of patients after surgery	Use QoR-40 to assess the quality of life of patients after surgery

*ERAS* enhanced recovery after surgery, *TAP* Transversus Abdominis Plane, *PCA* Patient controlled analgesia, *VAS* visual analogue scale, *QoR-40* the Quality of Recovery-40 Questionnaire

#### QoR-40 structure and scoring rules

The QoR-40 is a self-rated 40-item questionnaire used to assess the recovery of the quality of life. The questionnaire consists of five dimensions: emotional status (9 items), physical comfort (12 items), psychological support (7 items), physical independence (5 items), and pain (7 items).All the items are rated on a five-point scale ranging from 1 to 5. The initial point and conversion score of each item are calculated. Depending on the question, 5 points or 1 point may be the best answer. The best answers to positive questions are scored 5, while the best answers to negative questions are assigned the score of 1. The total score of the QoR-40 is the sum of the scores of all items. The score of each dimension is the sum of the total scores of the items in the corresponding dimension. The total score ranges from 40 to 200. The higher the score, the better is the quality of life [18].

### Data collection

On the Baseline day, the investigator briefly explained the purpose and significance of the study and the anonymity and confidentiality of the study data. The patients were then required to sign a written informed consent form, and they were asked to complete the QoR-40 to determine the Baseline health status before surgery. The patients also completed the QoR-40 on POD1, 3, 6, and 30 according to their actual situation. If required, the investigator provided the necessary assistance to the patient to complete the QoR-40. The patient demographic and perioperative data were collected simultaneously, including postoperative complications, albumin (Alb), hemoglobin (Hb), score of patient-generated subjective global assessment (PG-SGA), ASA grading, visual analog scale (VAS), type of surgery, first time off-bed activity, first time of flatus, time to removal of nasogastric tube, time of removal of urine catheter, length of postoperative hospital stay, hospitalization cost, and hospital readmission within 30 days. Postoperative complications were classified according to the Clavien-Dindo postoperative complications classification standard [19]. Discharge standard (based on the discharge standard of the General Hospital of Nanjing Military Region, China) was as follows: intestinal function recovery, oral intake of 70% of the preoperative intake level; no requirement for intravenous rehydration; no pain or pain can be effectively relieved by oral analgesics; ability to complete daily activities normally and to take care of themselves; and willingness to be discharged from the hospital.

### Statistical analysis

All statistical analyses, including data entry, descriptive statistical analysis, paired t-test, and analysis of variance (ANOVA), were performed using SPSS 25.0 software (IBM, Corp.). Measurement data were expressed as mean ± standard deviation (χ ± s). The measurement data were compared between the two groups by using t-test, while the chi-square test was used to compare count data. Statistical significance for all analyses was set at p < 0.05.

## Results

A total of 221 patients undergoing radical GC surgery were enrolled in this study. Of these patients, 21 patients could not complete the QoR-40 for various reasons and were excluded from the study. Finally, a total of 200 patients effectively completed the QoR-40, including 100 patients in the ERAS group and 100 patients in the Contr group. The demographic and basic clinical characteristics of the patients during the perioperative period are shown in Table 2. No significant difference was observed between the ERAS and Contr groups in gender, age, height, weight, body mass index (BMI), education level, and ASA grading for anesthesia (p = 0.083, 0.058, 0.963, 0.964, 0.416, 0.337, and 0.092, respectively). In terms of preoperative nutritional indicators, the comparison of Alb, Hb, and PG-SGA scores between the two groups showed no significant difference (p = 0.144, 0.169, and 0.133, respectively).In the ERAS group, there were 61 cases of laparoscopic surgery and 39 cases of robotic surgery, while in the control group, there were 17 cases of open surgery, 72 cases of laparoscopic surgery, and 11 cases of robotic surgery. Significant differences were observed between the two groups (p < 0.001) with regard to the type of surgery performed. The clinical outcomes of the patients are detailed in Table 3. Postoperative diet initiation time in the ERAS group was significantly earlier than that in the Contr group (p < 0.001). The removal of the nasogastric tube in the ERAS group was 1.79 ± 0.98 days after surgery, which was significantly earlier than that 3.94 ± 2.26 days after surgery in the Contr group (p < 0.001). The removal of the urine catheter in the ERAS group was also significantly earlier than that in the Contr group (p = 0.005). The length of hospital stay after surgery was 6.55 ± 1.43 days for the ERAS group and 12.79 ± 9.28 days for the Contr group, which was statistically significant (p < 0.001). There was significant difference in postoperative complications between the ERAS and Contr groups (p < 0.001). The hospital readmission rate of the two groups was 3% within 30 days. In terms of hospitalization cost, the average hospitalization cost of the ERAS group was 79,772.88 ± 25,816.84 yuan and that of the Contr group was 88,602.45 ± 28,288.84 yuan. A significant difference in hospitalization cost was observed between the two groups (p = 0.022).

**Table 2:** Participant characteristics (n = 200)

Characteristics	Control	ERAS	p-value
No. of patients	100	100	
Gender: Female/Male	34/66	46/54	0.083
Age (years)	54.59 ± 9.16	52.13 ± 11.57	0.058
Height (cm)	161.07 ± 12.88	161.14 ± 7.93	0.963
Weight (kg)	59.25 ± 15.84	59.16 ± 10.37	0.964
BMI (kg/m^2^)	25.73 ± 3.70	22.71 ± 3.09	0.416
Education: primary or below/secondary/high/university or above	36/31/19/14	27/35/24/14	0.337
ASA: I/II/III	3/62/35	3/74/23	0.092
Alb	36.25 ± 4.48	37.12 ± 3.88	0.144
Hb	115.87 ± 25.57	120.96 ± 26.59	0.169
PG-SGA	5.18 ± 3.72	4.42 ± 3.41	0.133
Resection range: total/subtotal gastrectomy	19/81	10/90	0.071
Operation: open/laparoscopic/robotic	17/72/11	0/61/39	< 0.001

*ERAS* enhanced recovery after surgery, *BMI* body mass index, *ASA* American society of anesthesiologists, *Alb *albumin, *Hb *hemoglobin, *PG-SGA* scored patient-generated subjective global assessment

**Table 3:** Clinical outcomes (n = 200)

Characteristics	Control	ERAS	P-value
No. of patients	100	100	
First flatus (hour)	82.63 ± 41.31	44.70 ± 16.32	< 0.001
Off-bed activity (hour)	43.82 ± 18.43	22.73 ± 10.60	< 0.001
Diet initiation (hour)	111.62 ± 49.40	51.14 ± 27.49	< 0.001
Removal of nasogastric tube(day)	3.94 ± 2.26	1.79 ± 0.98	< 0.001
Removal of urine catheter(day)	1.35 ± 0.76	1.11 ± 0.37	0.005
Postoperative complications: yes/no	32/68	10/90	< 0.001
Hospital stay after surgery (day)	12.79 ± 9.28	6.55 ± 1.43	< 0.001
Cost(yuan)	88,602.45 ± 28,288.84	79,772.88 ± 25,816.84	0.022
Hospital readmission (%)	3 (3.0%)	3 (3.0%)	1.000

*ERAS* enhanced recovery after surgery

**Fig. 1:**
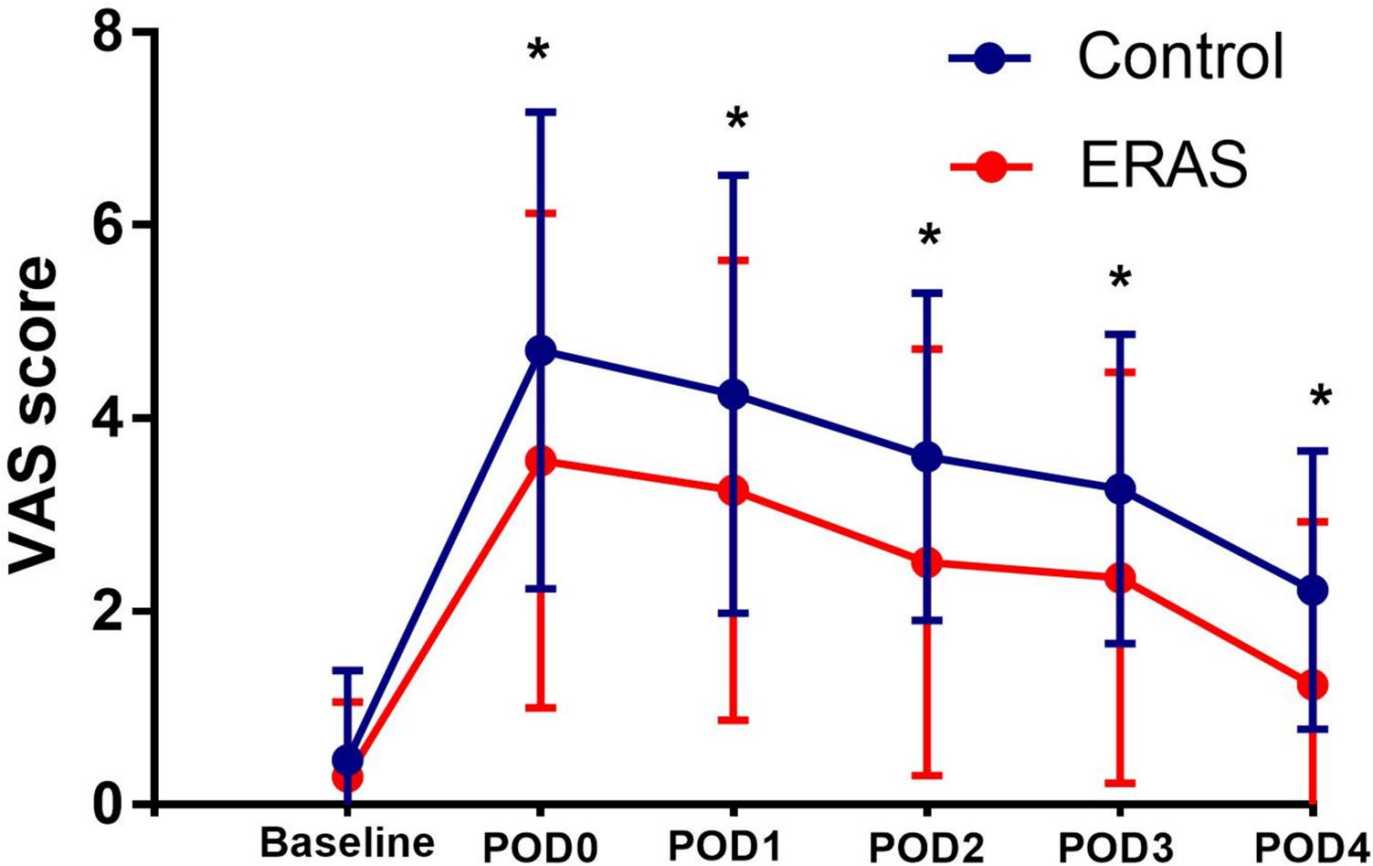
Changes (mean ± SD) in VAS score in ERAS group and Control group at different time. *p < 0.05. *VAS* visual analog scale, *ERAS* enhanced recovery after surgery, *Baseline* one day before surgery, *POD *postoperative day

The changes in VAS scores in the ERAS and Contr groups at each time point are shown in Table 4 and Fig. 1. There was no significant difference in VAS scores between the two groups at Baseline (p = 0.162). However, each time point after surgery, the VAS score of the ERAS group was significantly lower than that of the Contr group (p = 0.002, 0.003, < 0.001, 0.001, and < 0.001). Repeated-measures ANOVA revealed that the VAS scores at multiple time points showed significant differences between the ERAS and Contr groups (p < 0.001), indicating that patients in the ERAS group had less postoperative pain than those in the Contr group.

**Table 4:** Changes in VAS in ERAS group and control group at different assessment time points

Group	Baseline	POD0	POD1	POD2	POD3	POD4	Total
Control	0.46 ± 0.93	4.70 ± 2.47	4.25 ± 2.27	3.60 ± 1.70	3.27 ± 1.60	2.22 ± 1.44	
ERAS	0.29 ± 0.78	3.56 ± 2.56	3.26 ± 2.38	2.51 ± 2.21	2.35 ± 2.13	1.24 ± 1.69	
p-value	0.162	0.002	0.003	< 0.001	0.001	< 0.001	< 0.001

*VAS* visual analog scale, *ERAS* enhanced recovery after surgery, Baseline one day before surgery, POD postoperative day

**Fig. 2:**
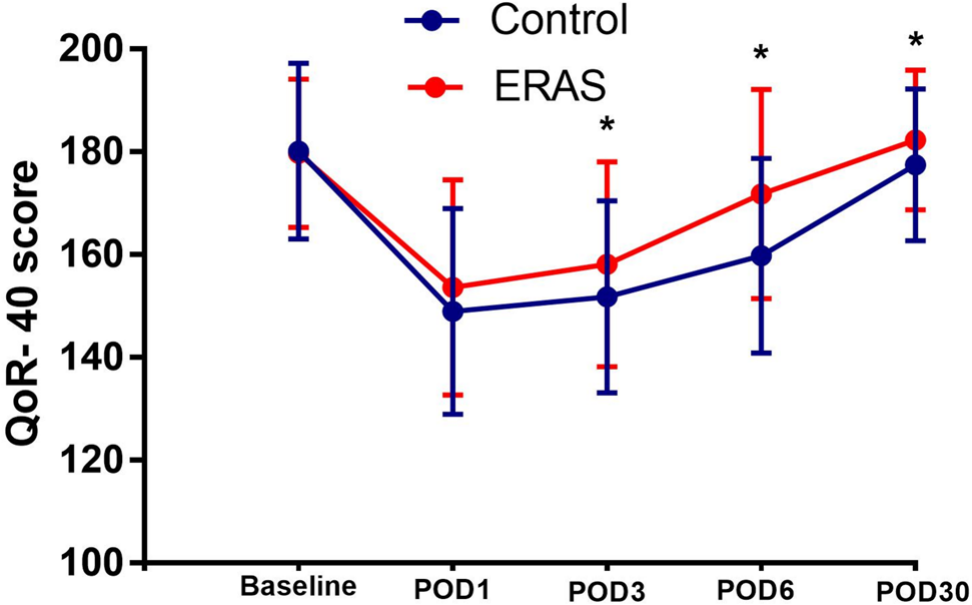
Changes (mean ± SD) in QoR-40 score in ERAS group and Control group at different time. *p < 0.05. *QoR-40 the Quality of Recovery-40 Questionnaire*, *ERAS* enhanced recovery after surgery, *Baseline* one day before surgery, *POD *postoperative day

Table 5 and Fig. 2 show the changes in the QoR-40 score in the ERAS and Contr groups at each time point. On the Baseline day, the QoR-40 score was 179.68 ± 14.46 in the ERAS group and 180.12 ± 17.12 in the Contr group, and no significant difference was observed between the two groups (p = 0.845). The QoR-40 scores of POD1, POD3, POD6, and POD30 in the ERAS group were 153.65 ± 20.92, 158.15 ± 19.98, 171.78 ± 20.37, and 182.28 ± 13.57, respectively, which were higher than those of the Contr group (148.92 ± 20.03, 151.76 ± 18.70, 159.83 ± 18.97, and 177.48 ± 14.74, respectively). No significant difference was observed between the two groups on POD1 (p = 0.105), while the QoR-40 scores of the remaining time points (POD3, POD6, and POD30) were significantly different (p = 0.018, < 0.001, and 0.019, respectively). A comparison of the QoR-40 scores at multiple time points between the ERAS and Contr groups by using repeated-measures ANOVA revealed significant differences (p = 0.006). The results of these time-dependent changes clearly showed that the patients in the ERAS group recovered faster than those in the Contr group from the perspective of patient-reported quality of recovery.

**Table 5:** Changes in QoR-40 score in ERAS group and control group at different assessment time points

Group	Baseline	POD1	POD3	POD6	POD30	Total
Control	180.12 ± 17.12	148.92 ± 20.03	151.76 ± 18.70	159.83 ± 18.97	177.48 ± 14.74	
ERAS	179.68 ± 14.46	153.65 ± 20.92	158.15 ± 19.98	171.78 ± 20.37	182.28 ± 13.57	
p-value	0.845	0.105	0.018	< 0.001	0.019	0.006

*QoR-40* the Quality of Recovery-40 Questionnaire, *ERAS* enhanced recovery after surgery, Baseline one day before surgery, *POD *postoperative day

In the ERAS group, the QoR-40 score at the Baseline was 179.68 ± 14.46 and decreased significantly on POD1 and POD3 to 153.65 ± 20.92 and 158.15 ± 19.98, respectively (both p < 0.001). On POD6, the QoR-40 score increased to 171.78 ± 20.37, but was still significantly lower than that at the Baseline (p < 0.001). On POD30, the QoR-40 score was 182.28 ± 13.57, which was not significantly different from that at the Baseline (p = 0.070) (Table 6).

**Table 6:** The QoR-40 scores in ERAS group at different assessment time points

ERAS	Baseline-POD1	Baseline-POD3	Baseline-POD6	Baseline-POD30
Mean ± SD	26.03 ± 18.57	21.53 ± 19.35	7.90 ± 19.22	− 2.68 ± 14.48
P-value	< 0.001	< 0.001	< 0.001	0.070

*QoR-40* the Quality of Recovery-40 Questionnaire, *ERAS *enhanced recovery after surgery, Baseline one day before surgery, *POD *postoperative day

**Fig. 3:**
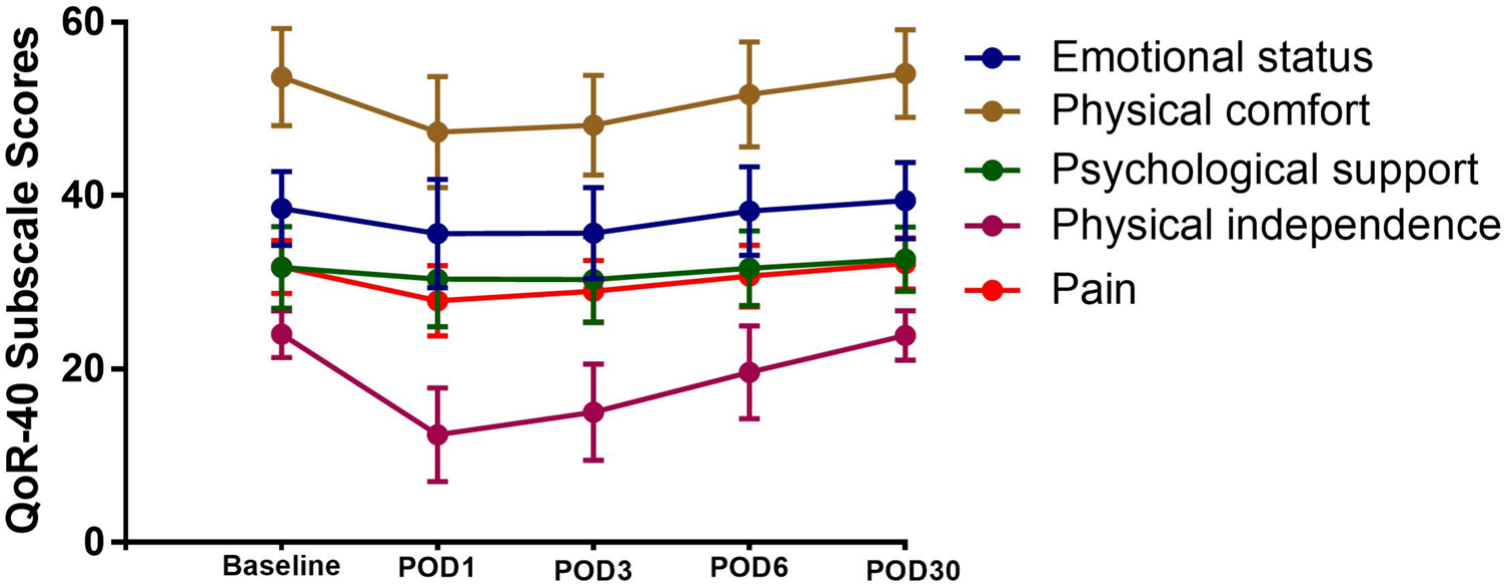
Changes (mean ± SD) in QoR-40 subscale scores in ERAS group at different time. *QoR-40* the Quality of Recovery-40 Questionnaire; *ERAS* enhanced recovery after surgery, *Baseline* one day before surgery, *POD* postoperative day

The scores of the five dimensions of the QoR-40 at each time point in the ERAS group are shown in Table 7 and Fig. 3. In all five dimensions, compared with the Baseline, the POD1 and POD3 scores were significantly decreased (all p < 0.05). On POD6, the scores of the two dimensions of “Physical comfort” and “Physical independence” returned to the Baseline level, while the scores of the dimensions of “Emotional status” “Psychological support” and “pain” were significantly lower than those at the Baseline level. On POD30, the scores of all five dimensions recovered to the baseline level; what is more, the score of the “Physical comfort” dimension (39.43 ± 4.38) was still significantly higher than the Baseline score (38.51 ± 4.28) (p < 0.005).

**Table 7:** The QoR-40 scores of the five dimensions before and after surgery in ERAS group

ERAS (max)	Baseline	POD1	POD3	POD6	POD30
Physical comfort (60)	38.51 ± 4.28	35.65 ± 6.26*	35.69 ± 5.24*	38.22 ± 5.12	39.43 ± 4.38*
Emotion state (45)	53.67 ± 5.62	47.32 ± 6.40*	48.14 ± 5.74*	51.68 ± 6.08*	54.08 ± 5.04
Physical independence (25)	31.73 ± 4.69	30.38 ± 5.48*	30.33 ± 4.94*	31.61 ± 4.30	32.67 ± 3.70
Psychological support (35)	24.00 ± 2.74	12.41 ± 5.41*	15.03 ± 5.55*	19.65 ± 5.39*	23.90 ± 2.86
Pain (35)	31.79 ± 3.06	27.88 ± 4.07*	28.95 ± 3.58*	30.70 ± 3.60*	32.19 ± 2.97

*QoR-40* the Quality of Recovery-40 Questionnaire, *ERAS* enhanced recovery after surgery, Baseline one day before surgery, *POD *postoperative day

*p < 0.05 (compared to baseline)

## Discussion

In recent years, the ERAS protocols in GC have matured, and many studies have reported its safety and effectiveness [20–22]. The safety and effectiveness indicators of ERAS protocols include objective indicators such as the length of hospital stay after surgery, hospitalization cost, and postoperative complications and subjective indicators such as the quality of life assessment questionnaire from the perspective of PROs. Presently, most studies are based on the results of objective indicators from doctor-reported outcomes, and there are few studies on the quality of life assessment from PROs. In the present study, the QoR-40 was used to investigate the postoperative recovery effect of ERAS protocols in GC from the perspective of patients’ subjective perception.

ERAS is a dynamic process, we do not have to implement all elements of ERAS, but the core elements should be implemented, as stated by Professor Kehlet, who proposed the ERAS concept [17]. The clinical practice of ERAS for GC in our center comprises 22 items including six core elements. Our present study showed no significant difference between the ERAS and Contr groups in terms of age, gender, height, weight, BMI, education, ASA grading for anesthesia, and nutrition. All parameters of the two groups were at the same baseline level before surgery. Compared to the Contr group, minimally invasive surgery with laparoscopic and robotic assistance was performed more in the ERAS group. The 2014 ERAS guidelines recommend to use minimally invasive surgery to shorten the incision length and reduce tissue trauma [16]. Some researchers have shown that minimally invasive surgery can significantly reduce the level of traumatic stress factors such as interleukin-6 after surgery [23]. A recent multicenter clinical study in China showed that ERAS combined with the laparoscopic approach achieves the same therapeutic effect as open surgery; does not increase the incidence of postoperative complications; shortens the time of early off-bed activity, early diet initiation, and first flatus time; and shortens hospital stay [24]. Our center adopted this opinion and has prioritized minimally invasive surgery in the ERAS protocols of GC. In terms of VAS scores, the ERAS group showed significantly lower VAS scores than those of the Contr group, which indicates that multimodal analgesia is effective in the ERAS group. This result is similar to that reported by Yamada et al. [25]. The ERAS group started recovery activities significantly earlier than the Contr group in terms of early off-bed activity, early removal of the nasogastric tube, early diet initiation, and first flatus time. There findings are consistent with those of previous studies. Compared to the traditional treatment group, the application of ERAS protocols can quickly restore the gastrointestinal function after surgery [26]. In the present study, there was no significant difference between the ERAS and Contr groups in terms of 30-day hospital readmission rates. Several studies have confirmed no significant difference between the ERAS and control groups in 30-day hospital readmission rates, which objectively demonstrates the safety of ERAS protocols for GC [27, 28]. In the present study, the average length of hospital stay after surgery in the ERAS group was 6.55 ± 1.43 days, which was significantly lower than that 12.79 ± 9.28 days of the Contr group. Sugisawa et al. [28] reported that the median hospital stay of the ERAS group after surgery was 8 days, which was significantly lower than that of the control group (*p* < 0.001). In terms of hospitalization cost, the ERAS group in the present study showed significantly lower hospitalization cost than the Contr group; a finding similar that found in the report of Wang et al. [29]. Li et al. [30] showed that the clinical practice of ERAS for GC by using laparoscopic surgery leads to quicker postoperative recovery and does not increase the rate of readmission and complications, thereby reducing hospital stay after surgery and the subsequent hospitalization cost. In general, our objective indicator-based results of ERAS clinical practice for GC are consistent with those of other centers, which demonstrates the safety and effectiveness of ERAS protocols from objective indicators.

The present study used the QoR-40 to investigate the effectiveness of ERAS protocols in GC from the perspective of PROs. Our previous study showed that despite cultural differences, the QoR-40 has acceptable validity, reliability, and responsiveness in assessing the health status of Chinese patients after surgery [15].The QoR-40 has been widely used to evaluate the quality of recovery after surgery [31, 32]. Jr et al. [33] used the QoR-40 to evaluate the effect size for the transversus abdominis plane infiltration on quality of postoperative recovery in patients undergoing laparoscopic gastric band surgery. Some researchers have used the QoR-40 to evaluate the quality of recovery after general anesthesia in patients undergoing laparoscopic cholecystectomy [34]. Therefore, the present study used the QoR-40 to evaluate the effect of ERAS protocols in GC. This study showed that the QoR-40 scores of the ERAS and Contr groups were the same on the Baseline day, which indicated that the two groups showed similar characteristics at the baseline level before surgery. Subsequently, the QoR-40 scores of the ERAS group were higher than those of the Contr group at each time point after the surgery. Except for POD1, significant differences in the QoR-40 scores were observed between the ERAS and Contr groups on POD3, POD6, and POD30. Repeated-measures ANOVA showed that the QoR-40 scores at multiple time points were significantly different between the two groups. According to the QoR-40 scores, which are based on PROs, the postoperative recovery of the ERAS group was found to be significantly better than that of the Contr group. Thus, the present study using the QoR-40 revealed the effectiveness of ERAS protocols in GC on the basis of the subjective perception of patients.

In the ERAS group, the QoR-40 score of patients with GC decreased significantly on POD1 and gradually recovered on POD3 and POD6. No significant difference in the QoR-40 score was observed between POD30 and Baseline. These results of time-dependent changes showed that the QoR-40 scores of the ERAS group gradually recovered over time after surgery, but did not return to the baseline level at the average hospital stay (POD6). On POD6, the scores of the two dimensions of “Physical comfort” and “Physical independence” in the QoR-40 returned to the baseline level, while the scores of the other three dimensions of “Emotional status,” “Psychological support,” and “Pain” were still significantly lower than the Baseline. On POD30, the scores of all the five dimensions of the QoR-40 recovered to the baseline level, but the score of the “Physical comfort” dimension was still significantly higher than the baseline level. Other researchers have used the QoR-40 to evaluate the quality of recovery after different types of surgery; however, their results were inconsistent with those of our present study for GC surgery. Wang et al. [35] showed that the QoR-40 scores of patients who underwent upper gastrointestinal surgery were significantly lower on POD1 and POD2 than on the Baseline day; a finding which was consistent with our research results. Shida et al. [32] used the QoR-40 in ERAS protocols in colorectal cancer to determine the postoperative recovery of patients, and their results showed that the QoR-40 score on POD6 had returned to the preoperative baseline level. Recently, Yin et al. [36] also obtained similar results in ERAS protocols in colorectal cancer combined with minimally invasive surgery, and in their study, the QoR-40 score returned to the preoperative baseline level on the day of discharge. Myles et al. [18] used the QoR-40 in studies on recovery after cardiac surgery and showed that the quality of life of patients did not return to baseline levels up to POD30. Poitras et al. [37] used the QoR-40 in patients who underwent joint replacement surgery and showed that compared to the baseline, the QoR-40 score did not show any significant difference even on POD1. Kobari et al. [38] studied patients who underwent robot-assisted partial nephrectomy under general anesthesia; in their study, the QoR-40 score decreased on POD1, but returned to the preoperative level on the average discharge day (3 ± 0.7 days). All these findings imply that different types of surgeries have different postoperative recovery conditions, which require a specific analysis for each surgery type. GC surgery is more traumatic, and therefore, postoperative recovery may take longer.

The present study has several limitations. First, this study is a single-center study, and prospective studies with multiple centers and larger sample sizes are needed in the future to verify the results of this study. Second, this study did not comply with the principle of completely randomized controlled trials. Thus, a certain degree of selection bias is inevitable in this study. The strength of this study is that it is the first study to use the QoR-40 to assess the quality of postoperative recovery of ERAS protocols in GC.

## Conclusion

From the results of this study, the following conclusions can be derived: (1) objective indicators confirm that ERAS protocols in GC are safe and effective and (2) according to the PROs, the postoperative recovery effect of ERAS protocols in GC is significantly better than that of the traditional treatment model, and that the patients discharged from the hospital on POD6 do not achieve recovery to the preoperative level.

## Data Availability

All data generated or analysed during this study are included in the article, further inquiries can be directed to the corresponding author.
